# What a picture selection task can tell us about scalar implicature processing? A neuroimaging investigation

**DOI:** 10.1098/rstb.2023.0496

**Published:** 2025-08-14

**Authors:** Tal Tehan, Einat Shetreet

**Affiliations:** ^1^ Sagol School of Neuroscience, Tel Aviv University, Tel Aviv 6139001, Israel; ^2^ Linguistics Department, Tel Aviv University, Tel Aviv 6139001, Israel

**Keywords:** Theory of Mind, executive functions, pragmatics, neurolinguistics, anterior cingulate, fMRI

## Abstract

Pragmatic inferences beyond the literal meaning of utterances occur with weak scalar expressions, such as ‘some’, which has a logical meaning of ‘some and possibly all’ and a pragmatic meaning of ‘some but not all’, derived through a scalar implicature (SI). Such processing is assumed to involve linguistic and extra-linguistic processes, including Theory of Mind and executive functions. Previous neuroimaging studies used truth value evaluation tasks to study SI processing, showing involvement of the left inferior frontal gyrus (IFG), left anterior middle frontal gyrus (MFG) and medial frontal gyrus (MeFG)/anterior cingulate cortex (ACC). This study used a picture selection task to better understand the role of these regions in SI processing. A region of interest analysis showed differences between a condition that promoted an implicature (with a weak scalar expression) and a condition that did not (with a strong scalar expression) only in the MeFG/ACC, also identified in a complementary whole-brain analysis (together with posterior activations). Based on the differences between processes related to truth value evaluation tasks and picture selection tasks, we propose that the IFG is linked to access to alternatives, the anterior MFG is associated with response-related processing, and the MeFG/ACC is associated with Theory of Mind.

This article is part of the theme issue ‘At the heart of human communication: new views on the complex relationship between pragmatics and Theory of Mind’.

## 1. Introduction

In day-to-day communication, we often have to derive pragmatic inferences in order to understand the intended meanings of utterances that go beyond what is literally conveyed. Understanding these non-literal meanings is important for successful communication. In his influential view of communication, Grice [1] suggested that speakers follow certain conversational guidelines, or maxims, to achieve successful communications, namely, to be truthful, informative, relevant and brief. Using these guidelines, listeners consider several factors, such as the context and the speaker’s knowledge, to derive the speaker’s intended meaning through pragmatic processing.

One well studied phenomenon of pragmatic interpretation is that of weak scalar terms, such as *some*, which receive their pragmatic meaning through a scalar implicature (SI). Traditionally, it is assumed that the logical meaning of weak scalars defines a lower boundary only (e.g. for *some*, this is ‘some and possibly all’) (e.g. [1–3]). Yet, typically, the meaning of weak scalars is pragmatically enriched, using SI, to exclude stronger/more informative scalars (e.g. for *some*, this is ‘some but not all’). For example, in the process of interpreting the sentence ‘Some of Clara’s cats are black’, we consider Grice’s maxim of quantity, which states that the speaker should provide as much information as needed but no less. That is, a knowledgeable speaker would use the most informative statement. If so, we infer that the speaker could not have stated that all of Clara’s cats are black. Therefore, an SI is derived, and this sentence is understood as some, *but not all*, of Clara’s cats are black.

Several studies using various methodologies in several languages confirm this assumption, showing that the pragmatically enriched meaning of *some* is favoured by adults (e.g. [4–7], although diversity across scales is reported [8,9]). The most commonly used task is a binary truth evaluation. This usually results in varying rates of responses adopting the pragmatic interpretation or the logical meaning (e.g. [10–13]). However, other tasks, including a picture selection task, show a clear dominance of responses adopting the pragmatic interpretation (e.g. [4,6,7,14]). It was suggested that the differential response pattern comes from the more complex decision-making process in the truth evaluation task, where participants have to consider whether to tolerate pragmatic violations or be charitable towards the speaker (e.g. [4,7]). In the picture selection task, participants simply adopt the speaker’s intended meaning.

Across tasks, SI processing is assumed to involve extra-linguistic high cognitive functions, such as Theory of Mind (ToM) or Executive Functions (EF). ToM is the ability to represent the mental states of others. As such, it is needed to calculate the meaning intended by speakers. The link between ToM and the derivation of intended meanings is empirically supported, as studies show correlations between rates of pragmatic responses in SI tasks and scores in ToM tests (e.g. [15,16]; although similarities in SI comprehension have been reported for neurotypicals and individuals with autism spectrum disorders assumed to have poorer ToM: for a review, see [17]). EF are a set of abilities used for control and coordination of cognition and behaviour. In pragmatics, EF are assumed to be used for manipulating information, overriding the initial literal meaning and its inhibition. This is empirically supported in studies showing lower rates of pragmatic responses in SI tasks under cognitive load (e.g. [11,18,19]) and correlations between the rates of pragmatic responses and EF capacity (e.g. [20], but see failure to show unique contribution of EF [15]).

Additional support for the involvement of extra-linguistic cognitive functions in SI processing comes from neuroimaging studies. In such studies, activations in brain regions outside the canonical language areas were observed. More specifically, activations during pragmatic processing were shown in brain regions that are conventionally linked to ToM (e.g. [21], although see fine-grained analysis [22–24]; also see [25] for context-based SI) or EF (e.g. [22,26]).

Here, we focus on Shetreet *et al.*’s study [22] because it used a design, with a truth evaluation task, that allowed a dissociation between two processes related to SIs: implicature generation and implicature mismatch. Implicature *generation* concerns the derivation of the pragmatically enriched interpretation of *some* as *some but not all*. This process is expected in all cases of SI derivation, regardless of the task. Regions associated with this process were identified by comparing between conditions in which implicature is assumed (i.e. sentences with the weak scalar *some*) and a condition in which no implicature should occur (i.e. sentences with the strong scalar *every*), showing activations in the left inferior frontal gyrus (IFG) (BA 47). *Implicature mismatch* occurs in contexts where *some* is logically true but pragmatically false (i.e. where underinformative statements are used in truth evaluation tasks). This process is considered to be related to response selection and decision-making. As discussed above, it is relevant to truth evaluation but not to a picture selection task. Regions associated with this process were identified by comparing between implicatures in a mismatched context (i.e. sentences with *some* presented with pictures in which all of the entities performed the activity mentioned in the sentence) and implicatures in a matched context (i.e. sentences with *some* presented with pictures in which only some of the entities performed the activity mentioned in the sentence), showing activations in the left anterior middle frontal gyrus (MFG) (BA 10) and medial frontal gyrus/anterior cingulate cortex (MeFG/ACC).

Now, let us consider the role of the regions identified in Shetreet *et al.* [22]. First, the left IFG was activated in the implicature conditions, regardless of the context (matched or mismatched). This region was also shown in another study of SI processing with unique activations when comparing pragmatic and semantic violations related to the some–all scale [27]. Furthermore, this part of the IFG is conventionally linked to semantic processing (see [28] for a meta-analysis). Therefore, it was argued in Shetreet *et al.* [22] that this region is linked to the computation of the implicature, possibly through a semantic component (see [29] for further discussion).

The two prefrontal regions, the MFG (BA 10) and the MeFG/ACC, were activated in Shetreet *et al.* [22] for the mismatched implicature condition. Activations in these regions, and specifically the MeFG/ACC, were also observed in other studies of pragmatic processing, including irony [30], relevance implicatures [31,32] and indirect requests [33]. More broadly, these regions are commonly associated with high cognitive functions, such as ToM (e.g. [34–36]) and EF (e.g. [37,38]). Shetreet *et al.* [22] argued that it is possible that these regions were identified as part of their general role in pragmatic processing, which is enhanced in the mismatched context. Given the complex decision-making in this context (compared with the matched context), they also suggested that the increased brain activation in these areas may come from this response selection process, as participants had to decide whether to apply charity or pragmatic tolerance, and choose between the logical and the pragmatic interpretations when determining the truth-value of the sentence.

The current study focuses on providing more nuanced identification of the role of these three regions in scalar implicature processing. To do so, we examined their involvement in a picture selection task, using both a region of interest (ROI) analysis based on functional activations taken from a truth evaluation task and a whole-brain analysis. The ROI allowed us to focus on regions that were previously linked to implicature generation and implicature mismatch.

We expect some processing differences between the two tasks, on a few dimensions. Concerning implicature generation, we expect similar processing of SI generation across tasks. More specifically, the more informative alternative (with the strong scalar) is expected to be taken into account and then negated. Yet, it is possible that implicature generation is facilitated in the picture selection task because the informative alternative is more readily accessed (i.e. through the contrasting picture). Concerning extra-linguistic processes, we expect the mentalizing of the speaker’s intention to occur in both tasks. Critically, however, processes related to decision-making are expected to be modulated in the picture selection task, because participants do not have to decide whether or not to apply charity or pragmatic tolerance. Furthermore, the unanimous response pattern in this task suggests that the logical interpretation is not truly considered.

In this study, we compare sentences that trigger implicatures, with the weak scalar (SOME condition) and sentences that do not trigger implicatures, with the strong scalar (ALL condition). Based on the functions discussed above, we expect a difference between the SOME and ALL conditions in the IFG because of the implicature generation. In the prefrontal regions, we expect differences between the conditions if these regions are involved in ToM processing. However, if these regions are linked to decision-making processing, we do not expect differences between them.

## 2. Methods

### (a) Participants

A total of 37 participants (18 females, mean age = 26.4 and range = 20–39 years) participated in the experiment. One additional participant was excluded owing to low accuracy rate in the control items (i.e. under 60% correct responses). All the participants were right-handed, had no neurological, hearing or language impairment and were Hebrew native speakers, which was their sole language. The protocol of this study was approved by the ethics committees at Sheeba Tel HaShomer Medical Center and Tel Aviv University. All the participants gave written informed consent and were monetarily compensated.

### (b) Stimuli and procedure

**Figure 1.**
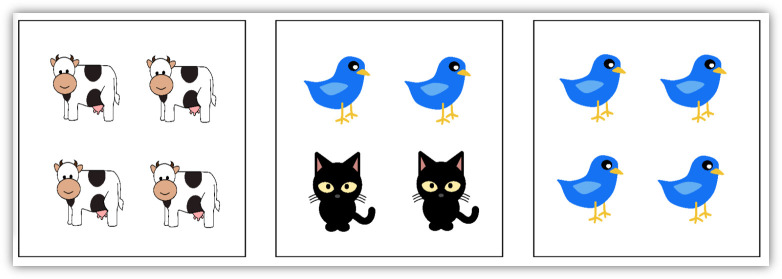
An example for one trial, presented with the sentence ‘*all*/*some of the animals are birds’* (translated here from Hebrew).

We used a picture selection task, adapted from Horowitz & Frank [39], in which participants looked at three pictures and were requested to choose the picture that matched a sentence that was presented simultaneously. The pictures depicted objects or animals that were either different items of the same category (e.g. animals: cows, birds and cats) or identical items with different colours (e.g. pink, red and blue balloons). Each picture included either four identical items (henceforth, all-pictures; e.g. right panel in figure 1) or two different pairs of items (henceforth, some-pictures; middle panel in figure 1). Each set of three pictures included one all-picture and one some-picture with one pair of the same items as the all-picture (e.g. for an all-picture with four birds, the some-picture included two birds and two cats; figure 1). The third picture showed items that did not appear in the other two pictures and was either an all-picture or a some-picture. The sentences included a quantifier, the equivalent of *some* in Hebrew (חלק, read *xelek*) or the equivalent of *all* (כל, read *kol*), which quantified the category presented in the pictures and then the specification of the subcategory (e.g. *some of the animals are birds*). Two conditions were defined based on the quantifier. The *SOME* condition included sentences with the weak quantifier and triggered an implicature, and the *ALL* condition included sentences with the strong quantifier and did not trigger an implicature. In both cases, the subcategory in the sentence referred to items that appeared in both the all-picture and the some-picture in the set (e.g. birds in figure 1). Additionally, filler items included either the weak or the strong quantifier (equally distributed across items) and referred to items that appeared only in one of the three pictures (e.g. *all of the animals are cows*).

We used an event-related design with 4 s trials. Pictures and auditory descriptions were presented simultaneously at the beginning of each trial to minimize the effect of memorization on participants’ response pattern (see [19]). Sentences were recorded by a female native speaker in a monotonous voice using a digital voice recorder and were processed using Audacity^®^ (https://audacityteam.org/). In order to ensure that participants listen to the entire sentence before making their selection, a tone, lasting 200 ms, was played 1800 ms after the onset of the audio, signalling the end of the sentence (all audio files were shorter than 1800 ms). Participants were instructed to respond only after hearing the tone and were allotted 1700 ms to do so, while the picture was still visible. They had to select the picture that best matched the sentence by clicking on one of the three buttons that corresponded with the picture location (i.e. left, middle and right). The last 300 ms of the trial presented a fixation cross, marking the end of the trial. Stimulus presentation was performed using the Psychtoolbox software.

We constructed four experimental lists, each containing 32 items of each experimental condition and 40 filler items, as well as rest trials with fixation cross. The items and the location of the correct picture were pseudorandomly ordered and counterbalanced across lists. Condition randomization was determined by optseq (http://www.freesurfer.net/optseq). The experiment was performed in a single run that lasted approximately 9 min. Participants performed a practice session on a computer prior to the magnetic resonance imaging (MRI) session. Practice trials included filler items only. The entire MRI session, including anatomical scans and additional functional scans, lasted approximately 1.5 h.

### (c) Data acquisition

MRI scans were conducted in a whole-body 3T Siemens Magnetom Prisma scanner (Siemens Medical Solutions, Erlangen, Germany) with a 64-channel head coil at the Strauss Center for Neuroimaging in Tel Aviv University. T1-weighted structural images were acquired using an MPRAGE pulse sequence with the following parameters: TR (Repetition Time) = 2530 ms, TE (Echo Time) = 2.88 ms, TI (Inversion Time) = 1100 ms, flip angle = 7° and 250 Hz px^−1^, with an isotropic voxel size of 1 mm³. Functional MRI was performed using a gradient-echo T2*-weighted echo planar imaging (EPI) interleaved sequence with 562 whole-brain images. The isotropic resolution was 2 mm^³^ (no gaps) with full brain coverage (with 66 axial slices per image). Scanning parameters were: field of view (FOV) = 200 mm, TR = 1000 ms, TE = 34 ms and flip angle = 60°, and a multiband acceleration factor of 6 without parallel imaging.

### (d) Data analysis

Data were pre‐processed with SPM12 software (Wellcome Department of Imaging Neuroscience, London, http://www.fil.ion.ucl.ac.uk). Images were slice-time corrected, realigned, coregistered to the subject’s anatomical MPRAGE scan, normalized to the brain template adopted by the International Consortium for Brain Mapping provided by SPM, and spatially smoothed using a Gaussian filter (8 mm kernel). Data of individual subjects were analysed using a generalized linear model (GLM) and high-pass filtered at 128 s. In the model, trial onsets were set to the simultaneous presentation of the picture and the audio with the duration of 4 s. We defined three regressors, one for the SOME condition, one for the ALL condition and one for the fillers. Head motion parameters were added as regressors. We defined a single contrast between the SOME and the ALL conditions. False discovery rate (FDR) correction was applied (*p* = 0.05) and a minimum cluster size of 100 voxels.

**Figure 2.**
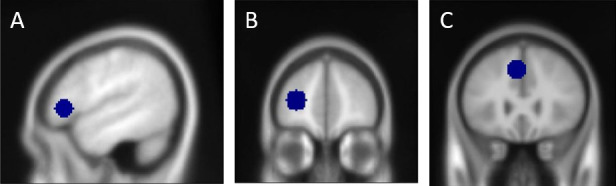
Regions of interest functionally defined based on a truth evaluation task from [22]: (A) left IFG, (B) anterior MFG and (C) MeFG/ACC. IFG, inferior frontal gyrus; MFG, middle frontal gyrus; MeFG/ACC, medial frontal gyrus/anterior cingulate cortex.

ROI analysis was performed using MarsBaR. We defined spheres (10 mm) around the peak coordinates taken from the main analysis in Shetreet *et al.* [22] (IFG: −48,29,2; anterior MFG: −30,50,10; and MeFG/ACC: −6,23,42; figure 2). Average beta-values were extracted for the two experimental conditions in the picture selection task (the SOME and ALL conditions). FDR correction for multiple comparisons was applied.

## 3. Results

### (a) In-scanner performance

In the two conditions with one possible response, the ALL condition and the fillers, accuracy rates were, as expected, very high (97.3%, s.d. = 0.03 and 93.9%, s.d. = 0.03, respectively). As predicted, for the SOME condition, an overwhelming majority of the responses adopted the pragmatically enriched interpretation (92.6%, s.d. = 0.06), such that all participants were classified as pragmatic responders. Still, there was a significant difference between the two experimental conditions (*t*(36) = 4.29, *p* < 0.001). Reaction times were not analysed because participants were specifically instructed to wait for the tone (indicating the end of the sentence) to give their responses.

### (b) Functional magnetic resonance imaging results

**Figure 3.**
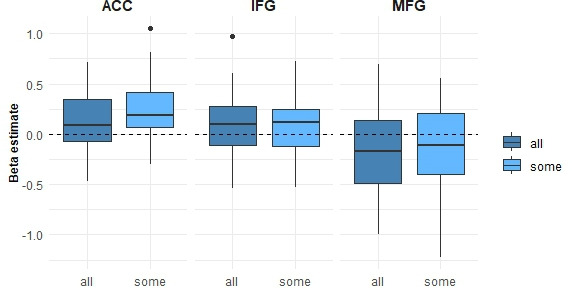
The extent of activations for SOME versus ALL conditions in each region of interest. ACC, anterior cingulate cortex; IFG, inferior frontal gyrus; MFG, middle frontal gyrus.

The ROI analysis allowed us to focus on specific regions that were defined *a priori*, increasing sensitivity to detect relevant effects. Here, we defined ROIs using a truth evaluation task taken from Shetreet *et al.* [22].^1^ Higher activations for the SOME condition than for the ALL condition were observed in the MeFG/ACC (*t*(36) = 3.1, *p* = 0.01; figure 3). No differences between the conditions were observed in the IFG (*t*(36) = 0.82, *p* = 0.41) or MFG (*t*(36) = 0.84, *p* = 0.41).

**Figure 4.**
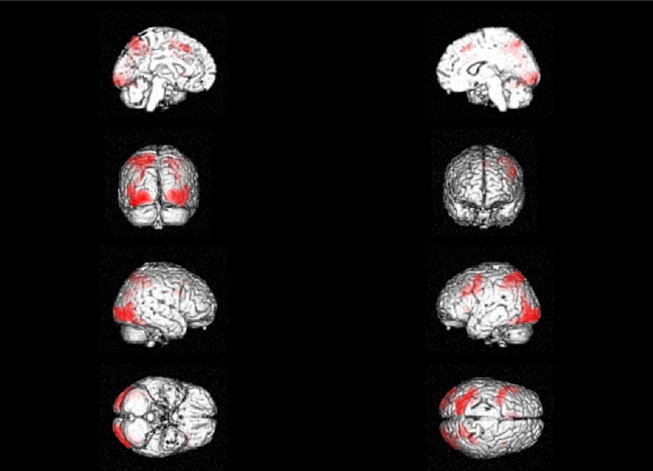
Areas activated in the comparison between the SOME condition and the ALL condition.

In a complementary whole-brain analysis, we compared the two conditions (SOME > ALL). This showed activations mostly in posterior regions, including bilateral clusters combining regions of the visual cortex and the superior parietal lobule/precuneus (BA 7). Activations were also observed in the posterior part of the MFG (unlike the anterior MFG observed in [22]; also in [41]). The only region identified in this analysis that was previously observed in the truth evaluation task was the MeFG/ACC (figure 4 and table 1).

**Table 1.** Areas of activation identified in the comparison between the SOME (implicature) and ALL (no implicature) conditions (SOME > ALL). MeFG/ACC, medial frontal gyrus/anterior cingulate cortex; MFG, middle frontal gyrus.

region	*x*	*y*	*z*	*K*	*t*_max_
left V1/visual association & superior parietal lobule/precuneus	−22	−94	−10	6024	8.09
	−32	−56	48		6.70
	−36	−82	−10		6.33
right V1/visual association & superior parietal lobule/precuneus	30	−90	-6	4436	8.05
	44	−76	-6		7.59
	40	−58	−16		6.55
left precentral gyrus	22	2	62	1721	5.67
	−42	2	36		5.03
	−38	6	50		4.45
left insula	−28	22	4	147	4.55
MeFG/ACC	−4	16	48	370	4.42
right posterior MFG (BA 46/9)	36	18	26	180	3.96
	40	8	32		3.45

## 4. Discussion

SI derivation is hypothesized to include several linguistic and extra-linguistic processes. Previous neuroimaging studies have identified a network of regions that underlies SI processing in a truth evaluation task [22]: the left IFG, for implicature generation and the anterior MFG and MeFG/ACC for response selection. The current study aimed to elucidate the roles of these regions using a different task. Unlike truth evaluation tasks, where both logical and pragmatic interpretations are observed, a picture selection task shows a clear dominance of pragmatic responses (92.6% of the responses in our experiment) [7,14]. Within the regions linked to SI processing in the truth evaluation task, differences between the implicature (SOME) and the no implicature (ALL) conditions in the picture selection task were observed only in the MeFG/ACC in both the ROI analysis and the whole-brain analysis.

This finding suggests that a picture selection task requires different cognitive resources from those observed in a truth evaluation task of the same pragmatic phenomenon in Shetreet *et al.* [22]. Most probably, both tasks require SI generation. However, the task settings and the post-interpretation response-related processes are different. In Shetreet *et al.* [7], we argue that participants in the picture selection task (among other tasks, named ‘participant-control’ tasks) are guided by the speaker’s intended meaning. In contrast, in truth evaluation tasks, participants evaluate a given sentence in the light of a certain state of affairs. This evaluation is guided by extra-linguistic considerations, beyond the speaker’s intended meaning. For example, participants have to consider whether to be pragmatically tolerant or to apply the charity principle. Importantly, these considerations are absent in the picture selection task. Thus, it seems that the truth evaluation task should place higher load on response-related processes than the picture selection task.

Indeed, the network of regions identified in the whole-brain analysis includes only a few prefrontal regions. Instead, regions related to visual and motor processing were identified. We note that trial modelling included the audiovisual presentation of the materials and the responses. We did this to capture both the implicature generation and the SI response-related processes. The extended activations in the visual cortex for the implicature condition suggest that more inspection of the pictures took place in this condition. This is in line with the ambiguous nature of implicatures, which allow two readings for the weak scalar. As the two alternative readings were available to the participants, they spent more time looking at the pictures. However, given the behavioural pattern of responses, and the lack of activation in the canonical decision-making regions, it seems that, despite its availability, the semantic reading was easily rejected. In other words, there was no conflict in the decision of which picture to select, leading to lower involvement in the response-related processes.

Of note are the posterior activations that included bilateral activations of the precuneus (BA 7) and the MeFG/ACC, which was also observed in previous studies (see more detailed discussion of this region below). These regions are repeatedly linked to ToM processing (e.g. [34,42]). A key assumption in prominent theories is that pragmatic processing involves calculating the speaker’s intention [43,1], suggesting that ToM plays a critical role in SI. As mentioned, we argue that the speaker’s intended meaning guides response selection in the picture selection task [7]. Thus, the picture selection task is expected to induce high load on ToM, comparable to that of the truth evaluation task.

### (a) *A priori* defined regions

Here, we discuss the three regions that we focused on in the ROI analysis. These regions were identified in [22] as related to implicature generation (left IFG) and to implicature mismatch (anterior MFG and MeFG/ACC).

#### (i) The left inferior frontal gyrus

This region is strongly linked with lexical–semantic processes (e.g. [28,44]). Shetreet *et al.* [22] argued that this area is related to the process of implicature generation because it was activated by sentences with weak scalars in both matched and mismatched contexts (and not by strong scalars). Because implicature generation is assumed to occur also in the picture selection task, we predicted a difference between the implicature and no implicature conditions. However, no such difference was observed in this task.

What then can explain the role of the left IFG in processing SIs? Based on the overall pattern of activations in this region in the two studies, such that the IFG was activated in [22] and also in [41], but not in the current study, we argue that this region is involved in accessing the lexical alternatives of the weak scalars (e.g. *all*). Most theories of SI agree that implicature generation includes, as a first step, the access to the relevant alternative, either by retrieving it from the lexicon or by construing it from the context (e.g. as in the case of ad hoc implicatures). Previous research has shown that making the alternatives salient and relevant promotes SI computation (e.g. [45,46]). In truth evaluation tasks, participants are not provided with alternatives and must retrieve the stronger alternative from the lexicon themselves. In contrast, in the picture selection task, the alternative readings of the sentence are presented by the pictures, making them more accessible and relevant. This should facilitate lexical access. Alternatives may even be construed from the context (i.e. the pictures), eliminating the need for lexical retrieval. If so, we can expect similar levels of activations for the implicature and no implicature conditions, where alternatives are not retrieved.

#### (ii) The anterior middle frontal gyrus

This region is linked to high cognitive functions. More specifically, activations in this region were observed in evaluation in yes/no judgement tasks [47], response inhibition [48] and strategy processes [49]. In Shetreet *et al.* [22], this area showed increased activations for the mismatched implicature context compared with the matched implicature context. Therefore, the authors attributed the role of this region to response selection. They argued that the mismatched condition had increased processing demands as both the logical and the pragmatic responses were appropriate, and, as noted earlier, participants have to decide whether to be charitable and/or tolerant towards the pragmatic violation.

The picture selection task used in this study is assumed to provide a more restrictive context for responses compared with the truth evaluation task, as suggested by its uniform response pattern. It is reasonable to assume that this task facilitates the decision-making process, making it easier for participants to select between the two possible readings of the weak scalar. Therefore, the lack of difference between the implicature and no implicature conditions in this study supports the suggestion that this region is engaged in response strategy.

#### (iii) The medial frontal gyrus/anterior cingulate cortex

This is the only region that was observed in the truth evaluation task in Shetreet *et al.* [22] and also activated in the picture selection task in the current study. Because both tasks are expected to include implicature generation, but differential task-related and response-related processes, our results suggest that the MeFG/ACC plays a crucial role in the generation process.

In terms of cognitive functions, this region was linked to both conflict monitoring [37,50,51] and ToM [34–36]. As suggested by Shetreet *et al.* [22], both of these cognitive functions are assumed to be involved in SI processing. A conflict between the two readings of weak scalars can occur in a truth evaluation task, as both readings are acceptable. This is indicated by the variation in the responses given in the task. ToM is required in SI derivation as the speaker’s knowledge, and cooperative intentions are considered.

What can the picture selection task tell us about the function of this region? As repeatedly mentioned, participants in this task almost unanimously choose the pragmatic interpretation. It is argued that this is because the task promotes interpretation based on the speaker’s intended meaning. Therefore, the conflict between the two readings of the weak scalar is assumed to be mitigated. If so, the increased activation in the implicature condition cannot be attributed to conflict monitoring. Instead, the role of this region in SI processing is probably linked to its role in ToM processing. As participants had to understand the speaker’s intentions in the implicature condition (and not in the no implicature condition), they utilized their mentalizing abilities supported by the MeFG/ACC.

All in all, we see differential response pattern and differential brain activation pattern when using a picture selection task compared with the patterns reported for a truth evaluation task (in [22]; also [41]). We acknowledge that the comparisons between the two tasks are not direct.^2^ Taking this into consideration, we assume these differences highlight the role of the identified regions in SI processing. We suggest that the left IFG is linked to the retrieval of the alternatives, the left anterior MFG is linked to response selection processes, and the MeFG/ACC is associated with ToM processing. This study highlights the need to use various tasks to test the same pragmatic phenomenon to better understand the cognitive mechanisms underlying its processing and their localization in the brain.

## Data Availability

Data can be accessed at [52].

## Footnotes

^1^
To ensure that ROIs were most relevant to our participants, we also used the results from a truth evaluation task performed by the same Hebrew-speaking participants [40]. This task followed the design in Shetreet *et al.* [22], including sentences with (Hebrew) weak or strong quantifiers presented with a picture that either matched, mismatched (for underinfomative sentences) or did not match the sentences. ROIs were extracted from the contrast between the mismatched implicature and a no implicature condition, following the main analysis in Shetreet *et al*. This resulted in three ROIs, similar to those identified in Shetreet *et al.*: left IFG (peak coordinates: −42,24,2), left anterior MFG (peak coordinates: −26,50,12) and MeFG/ACC (peak coordinates: −4,24,46). The results from ROIs defined on the same participants showed the same pattern as that found in the ROI based on the English-speaking sample from Shetreet *et al*., as differences between the SOME and ALL conditions were identified only for the MeFG/ACC (*t*(36) = 2.26, *p* = 0.044; and not for the IFG and MFG, *p* > 0.31).

^2^
Although the differential pattern between the studies can also be attributed to differences in the participants or the language, we note that the whole-brain analysis of the truth evaluation task within the same group of participants reported here showed similar results to those of Shetreet *et al.* [22]. Furthermore, when using this task as a functional localizer to define the ROIs, the same pattern of results appears (see note 1).
